# Prognostic value of the Controlling Nutritional Status (CONUT) score in cancer: an umbrella review of systematic reviews and meta-analyses

**DOI:** 10.3389/fnut.2026.1854644

**Published:** 2026-07-09

**Authors:** Xiaohua Wang, Jiyu Yang, Yingze Hou, Tong Wu, Xinru Wang, Xiaoyu Zhang, Xianchun Zhou

**Affiliations:** 1 Central Laboratory, The Affiliated Hospital of Yanbian University, Yanji, China; 2 Department of Oncology, Yanbian Hospital Affiliated to Yanbian University, Yanji, China

**Keywords:** cancer, Controlling Nutritional Status, outcomes, prognostic, umbrella review

## Abstract

**Background:**

The Controlling Nutritional Status (CONUT) score, combining serum albumin, total cholesterol, and lymphocyte count, has shown promise as a prognostic biomarker in various cancers, but a comprehensive assessment across different cancer types remains lacking.

**Methods:**

We systematically searched PubMed, Embase, Web of Science, and the Cochrane Database of Systematic Reviews from inception to October 2025 and identified 40 meta-analyses reporting 86 outcomes [7 non-statistically significant (*P* ≥ 0.05) and 79 statistically significant (*P* < 0.05)] related to the CONUT score and cancer prognosis. The corrected covered area (CCA) was computed to assess study overlap and evidence independence among pooled meta-analyses. The methodological quality of the included studies was evaluated using the AMSTAR, while the quality of the evidence was assessed using the GRADE. Pooled effect sizes, including hazard ratios (HRs), odds ratios (ORs), and relative risks (RRs), along with their 95% confidence intervals (CIs), were directly extracted. Heterogeneity was assessed using the I^2^ statistic and Cochran's Q test, and publication bias was evaluated using Egger's regression test.

**Results:**

A high CONUT score was suggested to be associated with poor prognosis across multiple cancer types. Among the 79 statistically significant outcomes, pooled HRs ranged from 1.19 to 3.94, RRs from 1.22 to 5.38, and ORs from 1.64 to 4.02. Most included meta-analyses (40%) focused on digestive system tumors. Moderate-quality evidence was identified for 10 outcomes. Notably, a high CONUT score was associated with increased postoperative mortality, with a pooled OR of 4.02 in patients with malignant tumors (MTs) and an RR of 5.38 in those undergoing gastrointestinal and hepatopancreatobiliary (GI-HPB) cancer surgery; both analyses showed no heterogeneity (I^2^ = 0%) and moderate-quality evidence.

**Conclusion:**

The available evidence, predominantly of low or very low quality (76/86 outcomes, 88.4%), suggests elevated CONUT scores are associated with poor prognosis across multiple cancer types. Moderate-quality evidence supports this association in specific contexts—including colorectal cancer, bladder cancer, and postoperative mortality in MTs and GI-HPB surgery—suggesting potential prognostic utility. However, given the overall low quality and substantial heterogeneity, routine clinical use of the CONUT score as an independent decision-making tool is not currently warranted.

**Systematic review registration:**

https://www.crd.york.ac.uk/PROSPERO/, identifier: CRD420261340659.

## 1 Introduction

Malnutrition is common among cancer patients and is strongly associated with poor survival outcomes across multiple malignancies (1–3). Elevated levels of inflammatory markers have also been identified as important predictors of poor cancer prognosis (4), as systemic inflammation may induce the invasion and proliferation of primary tumor cells, promote angiogenesis, accelerate tumor metastasis, and suppress antitumor immune responses (5–7). Furthermore, inflammatory status interacts with nutritional status and immune function, collectively exacerbating tumor progression (8). Therefore, identifying biomarkers that objectively and comprehensively reflect patients' nutritional and inflammatory status is crucial for prognostic stratification and clinical intervention in cancer patients.

The Controlling Nutritional Status (CONUT) score, a simplified tool integrating serum albumin, total cholesterol, and lymphocyte count (Table 1), was first proposed by Ignacio et al. (9). By simultaneously reflecting the host's nutritional and immune status (10), the CONUT score has gained recognition as a significant prognostic biomarker in the field of oncology. Numerous meta-analyses have investigated its correlation with long-term survival outcomes, such as overall survival (OS) and progression-free survival (PFS), across various cancer types. These studies have substantiated its predictive efficacy, particularly in gastric (11, 12), colorectal (13–15), and hepatocellular (16–18) carcinomas. Furthermore, initial evidence suggests its utility in predicting perioperative outcomes, including postoperative complications and mortality, in patients undergoing gastrointestinal and hepatopancreatobiliary (GI-HPB) surgical procedures for cancer (19–21).

**Table 1:** Standard CONUT (Controlling Nutritional Status) scoring system.

Parameters	Normal	Mild	Moderate	Severe
Serum Albumin (g/dL)	≥3.50	3.00–3.49	2.50–2.99	< 2.50
Score (Albumin)	0	2	4	6
Total Lymphocyte Count (/mL)	≥1,600	1,200–1,599	800–1,199	< 800
Score (Lymphocyte)	0	1	2	3
Total cholesterol (mg/dL)	≥180	140–179	100–139	< 100
Score (Cholesterol)	0	1	2	3
Total CONUT score	0–1	2–4	5–8	9–12

The total score ranges from 0 to 12 points. Higher total scores correspond to more severe nutritional impairment.

Despite the widespread interest in the prognostic significance of the CONUT score, the current body of evidence is notably fragmented, exhibiting substantial variability in cancer types, outcome definitions, methodological quality, and heterogeneity. A comprehensive synthesis across various cancer types and outcomes is lacking. Consequently, this study conducted a secondary synthesis of published pooled estimates from existing meta-analyses within an umbrella review framework, thereby systematically evaluating and summarizing the prognostic value of the CONUT score across various malignancies. Additionally, it aims to systematically evaluate the reliability and sources of heterogeneity within the evidence. The ultimate objective is to map the current evidence landscape and to provide evidence-based recommendations for optimizing future research trajectories.

## 2 Methods

### 2.1 Umbrella review methods

This study synthesized evidence from published systematic reviews and meta-analyses on the association between the CONUT score and cancer prognosis. We included only meta-analyses, excluding systematic reviews without quantitative synthesis, and conducted an umbrella review to evaluate the prognostic value of the CONUT score. We prospectively registered this umbrella review in PROSPERO (CRD420261340659, available at: https://www.crd.york.ac.uk/PROSPERO/).

### 2.2 Literature search

We searched PubMed, Embase, Web of Science, and the Cochrane Database of Systematic Reviews from their inception to October 2025 (last update date) to identify systematic reviews and meta-analyses investigating the association between the CONUT score and cancer prognosis. The search strategy was designed in accordance with PRISMA-S guidelines and structured around three core concepts: (1) neoplasms (using MeSH terms and free terms including “Neoplasms” [Mesh], cancer, tumor, malignancy); (2) the target biomarker (“Controlling Nutritional Status” OR “CONUT”); and (3) study design (“systematic review” OR “meta-analysis”). Two authors (WXH and YJY) independently performed the electronic search, screened titles and abstracts, and assessed full-text articles against the inclusion criteria. Any discrepancies during the screening process were resolved through discussion with a third author (ZXC). Additionally, we manually examined the reference lists of all included articles to identify potentially relevant studies that might have been missed in the database searches.

### 2.3 Inclusion and exclusion criteria

The studies included in this umbrella review were published meta-analyses that focused on the association between the CONUT score and prognostic outcomes in patients with various cancers. Eligible meta-analyses had to meet the following criteria: (1) the CONUT score was used as the primary exposure factor; (2) the association with at least one prognostic outcome was reported, including either long-term survival outcomes [such as OS, PFS, disease-free survival (DFS), recurrence-free survival (RFS), event-free survival (EFS), or cancer-specific survival (CSS)] or short-term perioperative outcomes (e.g., postoperative complications and postoperative mortality); and (3) effect measures [e.g., hazard ratios (HRs), odds ratios (ORs), or relative risks (RRs)] with their corresponding 95% confidence intervals (CIs) were provided.

To avoid overlap of primary studies, when multiple meta-analyses addressed the same cancer type, the same patient subgroup, and the same outcome, the study for inclusion was selected according to the following hierarchical criteria:

1. Recency of Evidence: If eligible meta-analyses were published more than 24 months apart, the most recently published study was selected.
2. Sample Size: If the publication dates fell within a 24-month window, the meta-analysis with the larger total sample size (number of participants) was selected.
3. Methodological Rigor: If the above criteria were insufficient for selection, priority was given to the meta-analysis with the higher methodological quality score as assessed by the AMSTAR tool.

We excluded non-English publications, animal studies, and laboratory-based basic research. Potential unadjusted patient overlap may exist across subgroup analyses within a single included meta-analysis, which may introduce minor statistical bias.

### 2.4 Corrected covered area (CCA)

We initially selected eligible meta-analyses based on recency, sample size, and AMSTAR score to ensure timeliness and methodological quality. Nevertheless, high-quality meta-analyses may share overlapping primary studies, thereby compromising the independence of the pooled evidence. We therefore calculated the corrected covered area (CCA) according to Pieper et al. (22) using the final 40 included meta-analyses. For citation matrix construction, each unique primary study was counted once per meta-analysis, regardless of multiple subgroup analyses within the same meta-analysis. Accordingly, the total number of citations (N) represented the sum of all cross-meta-analysis repetitions of primary studies while excluding within-meta-analysis duplicates. The CCA was calculated using the following formula:


$$\begin{array}{l} CCA=\frac{N-r}{c\times r-r} \end{array}$$

where N is the total number of citations (i.e., the sum of all references across meta-analyses, counting duplicates), r is the number of unique primary studies, and c is the number of included meta-analyses. We interpreted CCA values as follows (22): 0–5% = slight overlap; 6–10% = moderate overlap; 11–15% = high overlap; and >15% = very high overlap. A lower CCA indicates greater independence of the evidence base. For cancer categories with high CCA values ( ≥ 11%), we interpreted the findings with caution and explicitly qualified the corresponding conclusions, as formal sensitivity analyses excluding overlapping primary studies were not feasible: the included meta-analyses reported only aggregated summary statistics without providing primary study-level effect size data.

### 2.5 Data extraction

Researchers WXH and YJY independently extracted data from all eligible studies, covering the following items: (1) the name of the first author; (2) the year of publication; (3) the stratification of tumor types; (4) endpoints related to survival; (5) the number of studies included in each analysis; (6) the total number of participants in each included study; and (7) the pooled effect size estimates, including HR, RR, and OR, along with their corresponding 95% CIs. Additionally, we also collected methodological parameters of the meta-analyses, such as the type of effect model employed (fixed-effect vs. random-effects), the I^2^ heterogeneity statistic, the *P*-value from Cochran's Q heterogeneity test, and any indication of publication bias, determined either through Egger's linear regression test or visual inspection of funnel plots. For meta-analyses that included dose-response analysis, the *P*-value for non-linear associations was also extracted. All discrepancies identified between the two reviewers were adjudicated by a third independent researcher (ZXC) to ensure data accuracy.

### 2.6 Assessment of methodological quality of included studies and quality of evidence

The methodological quality of the included studies was rigorously assessed using the 11-item AMSTAR (A Measurement Tool to Assess Systematic Reviews). The original 11-item version of AMSTAR was chosen due to its prevalent reporting within the included meta-analyses, thereby enhancing the feasibility of cross-study comparisons. As a fully validated tool, AMSTAR is specifically designed to evaluate the methodological rigor of systematic reviews and meta-analyses (23, 24). To ensure transparent and credible evidence grading in this umbrella review, we applied the GRADE approach to assess the certainty of evidence for all included outcomes and classified it into four levels (high, moderate, low, and very low). Because all included evidence originated from meta-analyses of observational studies, the initial certainty level for each outcome was set to “low” rather than to “high” (the latter being reserved for randomized controlled trials).

Evidence was downgraded according to the five GRADE domains: (1) risk of bias, assessed using AMSTAR scores, with scores < 8 indicating serious methodological limitations; (2) inconsistency, defined as I^2^ > 50% reflecting substantial between-study heterogeneity; (3) indirectness, assessed based on misalignment between the study population, study design, and the core research question; (4) imprecision, determined by the 95% confidence interval crossing the null or by including clinically insignificant effects, or by a *P* value > 0.001 for the effect estimate; and (5) publication bias, assessed using Egger's test (*P* < 0.05 indicating significant small-study effects) or asymmetric funnel plots. Evidence could be upgraded based on three factors: (1) large effect size (HR, OR, or RR > 2), (2) presence of a dose-response relationship, or (3) adequate statistical adjustment for all known confounding factors. No outcome in this review met the criteria for upgrading based on a dose-response relationship or adequate confounding adjustment.

For outcomes where Egger's test results were not reported (i.e., “NA” in the original meta-analyses), publication bias was considered “not assessable” rather than automatically classified as “undetected” or “strongly suspected”. In such cases, no downgrade for publication bias was applied based solely on the absence of assessment; however, this limitation was noted in the evidence summary. Only when funnel plots were clearly asymmetric, or when the original authors explicitly noted suspicion of publication bias, was this domain considered a serious concern.

The final GRADE rating, domain-specific judgments, and detailed justifications for each outcome are fully presented in Supplementary Table S4.

### 2.7 Data analysis

The pooled effect sizes, including HRs, ORs, and RRs, along with their corresponding 95% CIs, were directly extracted from the aggregated results of the primary meta-analyses incorporated in this review. We utilized the statistical model—either fixed-effects or random-effects—chosen by the authors of the primary meta-analyses, which was determined based on the outcomes of their heterogeneity assessments. Consequently, this study synthesized and appraised existing quantitatively synthesized evidence, rather than re-pooling raw data from the primary studies.

## 3 Results

**Figure 1:**
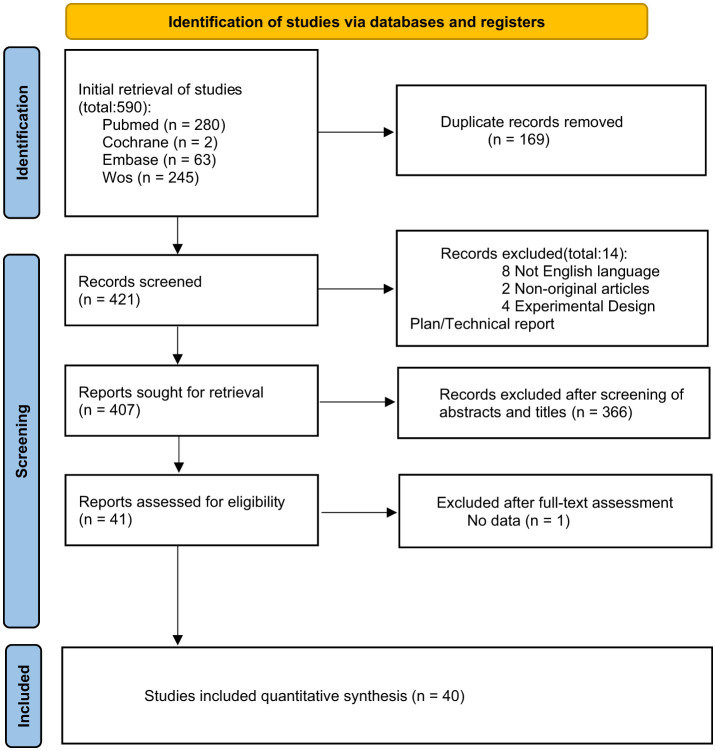
Flowchart depicting the literature search and study selection process for this umbrella review. Initial retrieval from PubMed (*n* = 280), Cochrane (*n* = 2), Embase (*n* = 63), and WoS (*n* = 245) yielded 590 records. After removing 169 duplicates, 421 records were screened, with 14 excluded (8 non-English, 2 non-original, 4 experimental design/technical reports). Among 407 sought reports, 366 were excluded after screening of abstracts and titles, 1 after full-text assessment of 41 reports, and 40 studies were finally included in quantitative synthesis.

**Figure 2:**
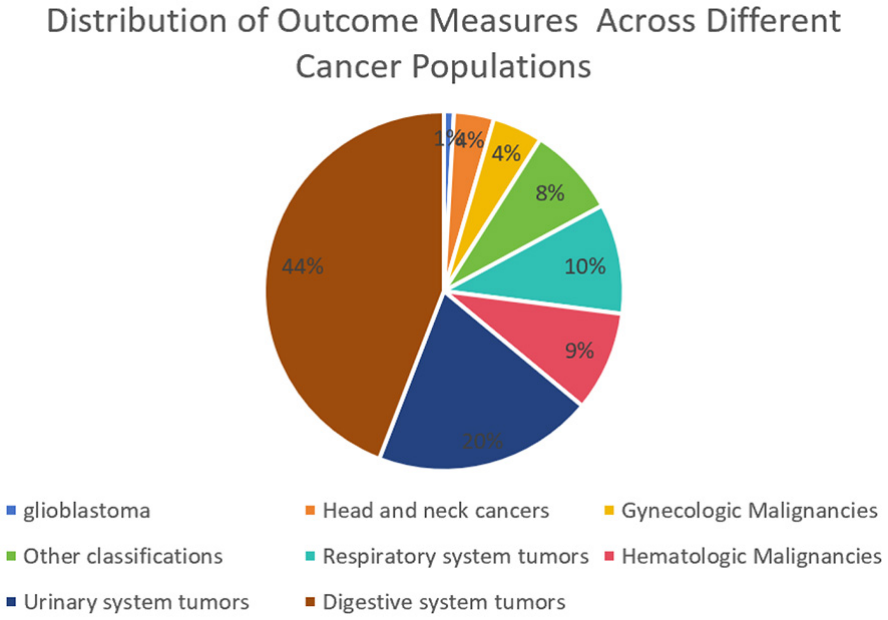
Pie chart showing the distribution of outcome measures across different cancer populations. Digestive system tumors account for the largest proportion (44%), followed by urinary system tumors (20%), respiratory system tumors (10%), hematologic malignancies (9%), other classifications (8%), gynecologic malignancies (4%), head and neck cancers (4%), and glioblastoma (1%).

**Figure 3:**
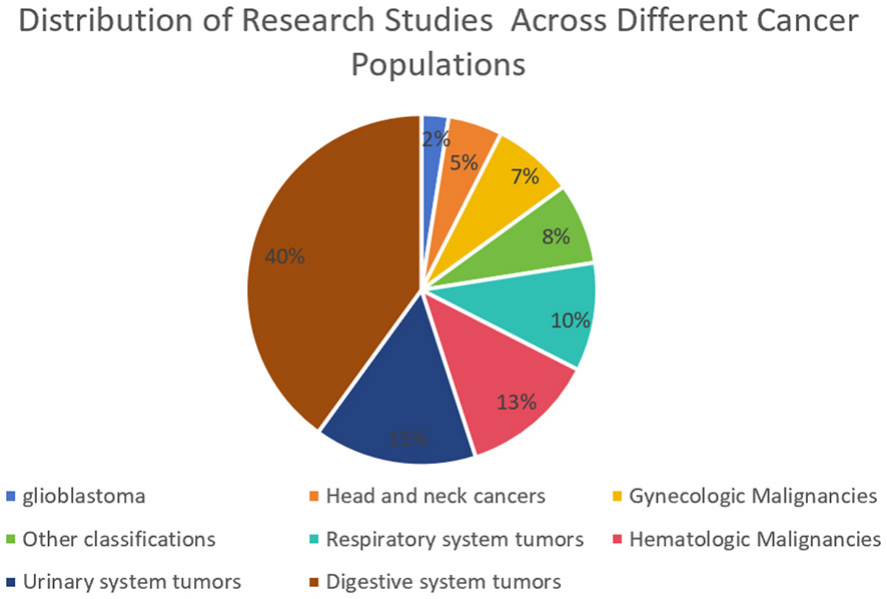
Pie chart showing the distribution of research studies across different cancer populations. Digestive system tumors account for the largest proportion (40%), followed by urinary system tumors (15%), hematologic malignancies (13%), respiratory system tumors (10%), other classifications (8%), gynecologic malignancies (7%), head and neck cancers (5%), and glioblastoma (2%).

The comprehensive literature search and screening process was illustrated in Figure 1. Of the 590 initial records, 40 meta-analyses met the inclusion criteria, encompassing 111 outcome measures (Figure 2). Among these outcomes, only OS and PFS for head and neck cancer were based on adjusted estimates. After applying the predefined inclusion criteria, 86 outcome measures were retained, including seven non-statistically significant indicators (*P* ≥ 0.05) and 79 statistically significant indicators (*P* < 0.05) (Supplementary Table S2). Among the 79 statistically significant outcomes, the pooled HRs ranged from 1.19 to 3.94, RRs from 1.22 to 5.38, and ORs from 1.64 to 4.02. However, the substantial heterogeneity in effect measures across studies warrants cautious interpretation. The meta-analyses mainly focused on cancers of the digestive system (*n* = 16), followed by those of the urinary system (*n* = 6), hematological malignancies (*n* = 5), and respiratory system cancers (*n* = 4). Other studies included gynecological (*n* = 3) and head and neck (*n* = 2) cancers, along with one study on glioblastoma. Three additional studies focused on cancers not categorized by specific organ systems (see Figure 3 for the distribution of cancer types in the included studies).

This study simultaneously included long-term survival outcomes and short-term perioperative outcomes for analysis: the former included OS, DFS, PFS, RFS, EFS, and CSS; the latter covered postoperative complications and 30-day postoperative mortality, which were used to assess the impact of nutritional status on surgical safety. The results indicated that a high CONUT score was not only significantly associated with poor prognosis in patients with various cancers but also associated with an increased risk of perioperative adverse events. However, the evidence supporting this conclusion was mostly rated as “low” or “very low” quality, and more high-quality original studies are needed for verification.

### 3.1 Outcomes supported by moderate-quality evidence

Ten outcome measures provided moderate-quality evidence across multiple cancer types and clinical contexts.

#### 3.1.1 For long-term survival

Colorectal cancer (CRC): Elevated CONUT score was associated with poorer OS and CSS, showing low heterogeneity (I^2^ = 34% for OS and 0% for CSS) (13, 15).

Esophageal cancer (EC): A high CONUT score was associated with poorer CSS, with no heterogeneity (I^2^ = 0%) (25).

Overall gastrointestinal (GI) cancers: Moderate-quality evidence supported associations with OS and CSS, both with no heterogeneity (I^2^ = 0%) (19).

Bladder cancer (BC): Elevated CONUT score was associated with worse OS and RFS, with no heterogeneity (I^2^ = 0%) (26).

**Figure 4:**
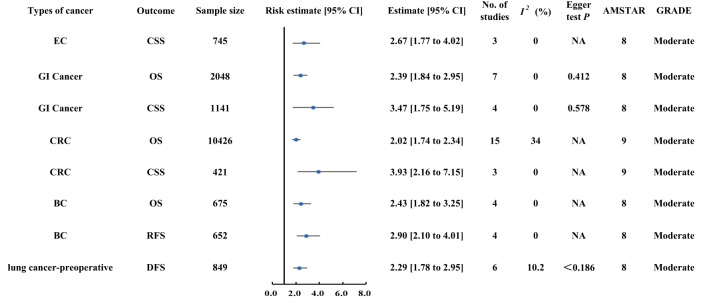
Forest plot for pooled associations between elevated CONUT score and long-term survival across moderate quality outcomes. Rows list cancer subtype, survival endpoint, sample size, study count, I^2^, Egger's P, AMSTAR score and GRADE rating. CI, confidence interval; AMSTAR, a measurement tool to assess systematic reviews; I^2^, inconsistency index (I^2^ statistic); Egger test, Egger's regression test; GRADE, grading of recommendations assessment, development, and evaluation; NA, not available; OS, overall survival; DFS, disease-free survival; RFS, recurrence-free survival; CSS, cancer-specific survival; EC, esophageal cancer; GI Cancer, gastrointestinal cancer; CRC, colorectal cancer; BC, bladder.

Preoperative lung cancer: A high CONUT score was associated with poorer DFS (I^2^ not reported), representing the highest-quality finding among respiratory analyses (27) (Supplementary Table S2, Figure 4).

#### 3.1.2 For postoperative mortality

In patients with malignant tumors (MTs), a high CONUT score was associated with postoperative mortality (pooled OR = 4.02) (28).

**Figure 5:**
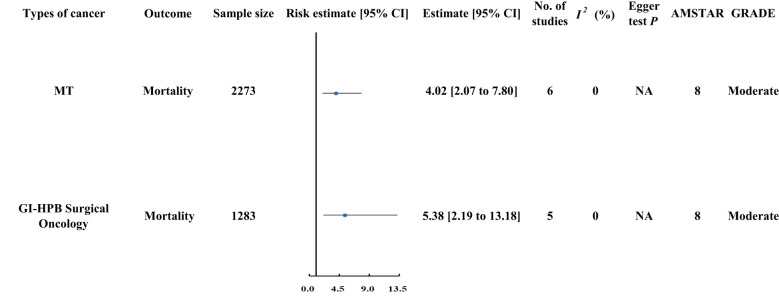
Forest plot for pooled associations between elevated CONUT score and postoperative mortality across moderate quality outcomes. Rows list cancer subtype, mortality endpoint, sample size, study count, I^2^, Egger's P, AMSTAR score, and GRADE rating. CI, confidence interval; AMSTAR, a measurement tool to assess systematic reviews; I^2^, inconsistency index (I^2^ statistic); Egger test, Egger's regression test; GRADE, grading of recommendations assessment, development, and evaluation; NA, not available; GI-HPB Surgical Oncology, gastrointestinal and hepatopancreatobiliary surgical oncology; MT, malignant tumors.

In patients undergoing GI-HPB cancer surgery, a high CONUT score was associated with postoperative mortality (pooled RR = 5.38) (20). Notably, both analyses showed no between-study heterogeneity (I^2^ = 0%) (Supplementary Table S2, Figure 5).

### 3.2 Outcomes supported only by low or very low quality evidence

Of the 79 statistically significant outcomes, only 10 were graded as moderate quality, whereas the remaining 69 outcomes (87.3% of all significant findings) were judged to be of low or very low certainty. Notably, elevated CONUT scores were consistently correlated with unfavorable survival outcomes across most major cancer types.

#### 3.2.1 For long-term survival

##### 3.2.1.1 Digestive system tumors

**Figure 6:**
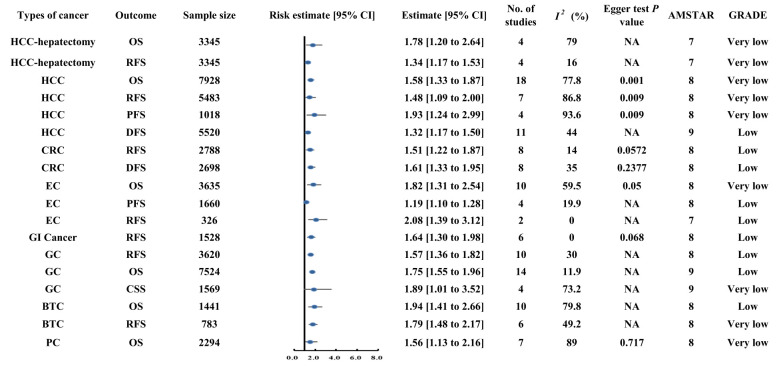
Forest plot for pooled associations between elevated CONUT score and long-term survival across digestive system malignancies supported by low or very low quality evidence. Rows list cancer subtype, survival endpoint, sample size, study count, I^2^, Egger's P, AMSTAR score, and GRADE rating. CI, confidence interval; AMSTAR, a measurement tool to assess systematic reviews; I^2^, inconsistency index (I^2^ statistic); Egger test, Egger's regression test; GRADE, grading of recommendations assessment, development, and evaluation; NA, not available; OS, overall survival; PFS, progression-free survival; DFS, disease-free survival; RFS, recurrence-free survival; CSS, cancer-specific survival; HCC, hepatocellular carcinoma; EC, esophageal cancer; GI Cancer, gastrointestinal cancer; CRC, colorectal cancer; GC, gastric carcinoma; PC, pancreatic cancer; BTC, biliary tract cancer.

Hepatocellular carcinoma (HCC) (16–18), biliary tract cancer (BTC) (29, 30), pancreatic cancer (PC) (31), and gastric cancer (GC) (12, 21, 32) all showed significant associations with poorer OS, PFS, DFS, RFS, or CSS. However, evidence quality was low to very low, and heterogeneity was moderate to high (I^2^ up to 93.6%) (Supplementary Table S2, Figure 6).

##### 3.2.1.2 Urinary system tumors

**Figure 7:**
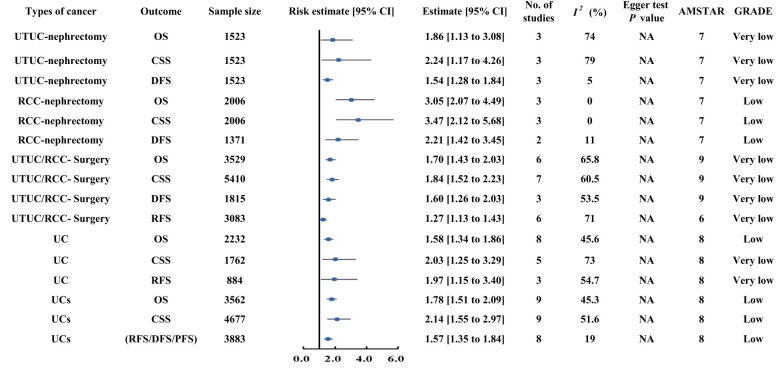
Forest plot for pooled associations between elevated CONUT score and long-term survival across urological malignancies supported by low or very low quality evidence. Rows list cancer subtype, survival endpoint, sample size, study count, I^2^, Egger's P, AMSTAR score, and GRADE rating. CI, confidence interval; AMSTAR, a measurement tool to assess systematic reviews; I^2^, inconsistency index (I^2^ statistic); Egger test, Egger's regression test; GRADE, grading of recommendations assessment, development, and evaluation; NA, not available; OS, overall survival; PFS, progression-free survival; DFS, disease-free survival; RFS, recurrence-free survival; CSS, cancer-specific survival; RCC, renal cell carcinoma; UTUC, upper tract urothelial carcinoma; UC, urological cancer; UCs, urological cancers.

In upper tract urothelial carcinoma (UTUC) (33–36), renal cell carcinoma (RCC) (33–36), and urothelial carcinoma (UC) (37), high CONUT scores predicted worse survival. However, most outcomes were of very low quality with moderate-to-high heterogeneity (Supplementary Table S2, Figure 7).

##### 3.2.1.3 Hematologic malignancies

**Figure 8:**
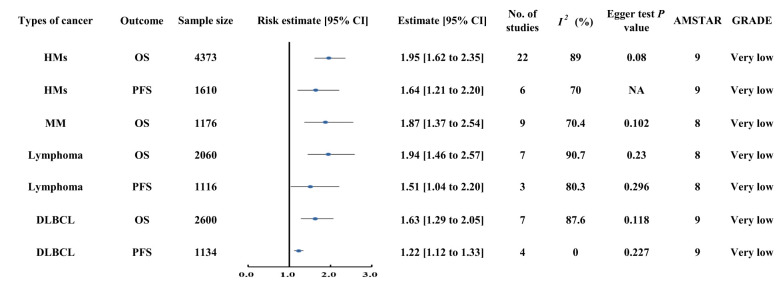
Forest plot for pooled associations between elevated CONUT score and long-term survival across hematological malignancies supported by very low quality evidence. Rows list cancer subtype, survival endpoint, sample size, study count, I^2^, Egger's P, AMSTAR score, and GRADE rating. CI, confidence interval; AMSTAR, a measurement tool to assess systematic reviews; I^2^, inconsistency index (I^2^ statistic); Egger test, Egger's regression test; GRADE, grading of recommendations assessment, development, and evaluation; NA, not available; OS, overall survival; PFS, progression-free survival; HMs, hematological malignancies; MM, multiple myeloma; DLBCL, diffuse large B-cell lymphoma.

In overall hematological malignancies (HMs) (38, 39), multiple myeloma (MM) (40), lymphoma (41), and diffuse large B-cell lymphoma (DLBCL) (42), high CONUT scores were associated with shorter OS and PFS. However, all findings were of very low quality with marked heterogeneity (I^2^ up to 90.7%) (Supplementary Table S2, Figure 8).

##### 3.2.1.4 Respiratory system tumors

**Figure 9:**
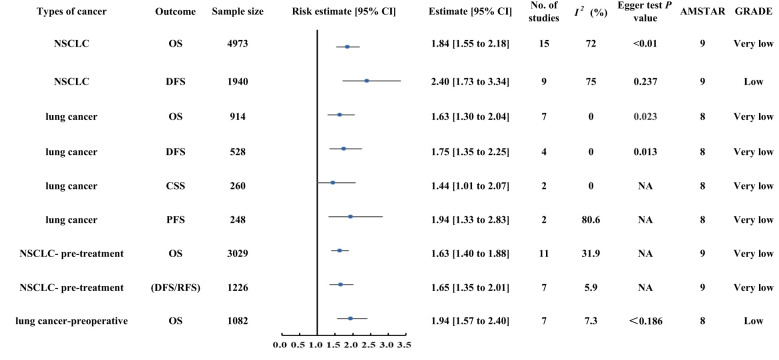
Forest plot for pooled associations between elevated CONUT score and long-term survival across respiratory system tumors supported by low or very low quality evidence. Rows list cancer subtype, survival endpoint, sample size, study count, I^2^, Egger's P, AMSTAR score, and GRADE rating. CI, confidence interval; AMSTAR, a measurement tool to assess systematic reviews; I^2^, inconsistency index (I^2^ statistic); Egger test, Egger's regression test; GRADE, grading of recommendations assessment, development, and evaluation; NA, not available; OS, overall survival; PFS, progression-free survival; DFS, disease-free surviva; RFS, recurrence-free survival; CSS, cancer-specific survival; NSCLC, non-small cell lung cancer.

In non-small cell lung cancer (NSCLC) (43, 44) and overall lung cancer (45, 46), high CONUT scores correlated with inferior OS, DFS, and PFS. However, evidence quality was mostly very low (Supplementary Table S2, Figure 9).

##### 3.2.1.5 Gynecological malignancies

**Figure 10:**
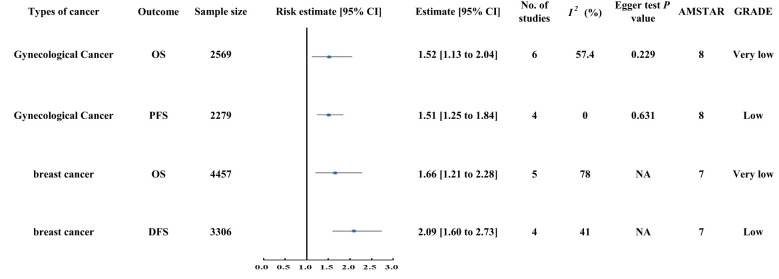
Forest plot for pooled associations between elevated CONUT score and long-term survival across gynecological malignancies supported by low or very low quality evidence. Rows list cancer subtype, survival endpoint, sample size, study count, I^2^, Egger's P, AMSTAR score, and GRADE rating. CI, confidence interval; AMSTAR, a measurement tool to assess systematic reviews; I^2^, inconsistency index (I^2^ statistic); Egger test, Egger's regression test; GRADE, grading of recommendations assessment, development, and evaluation; NA, not available; OS, overall survival; PFS, progression-free survival; DFS, disease-free surviva.

In gynecological cancers (47) and breast cancer (48, 49), elevated CONUT scores were associated with poorer OS and PFS. All these associations were supported by very low to low quality evidence. Notably, the OS analysis in breast cancer showed high heterogeneity (I^2^ = 78%) (Supplementary Table S2, Figure 10).

##### 3.2.1.6 Head and neck cancer (HNC)

**Figure 11:**
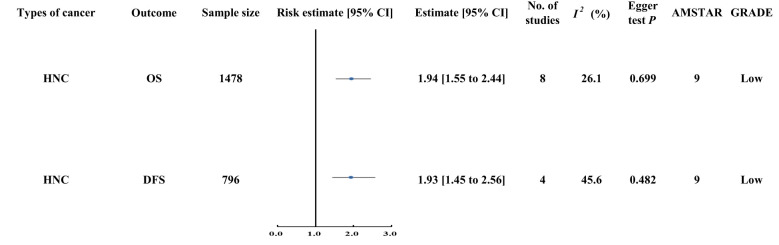
Forest plot for pooled associations between elevated CONUT score and long-term survival in head and neck cancer supported by low quality evidence. Rows list cancer subtype, survival endpoint, sample size, study count, I^2^, Egger's P, AMSTAR score, and GRADE rating. CI, confidence interval; AMSTAR, a measurement tool to assess systematic reviews; I^2^, inconsistency index (I^2^ statistic); Egger test, Egger's regression test; GRADE, grading of recommendations assessment, development, and evaluation; NA, not available; OS, overall survival; DFS, disease-free survival; HNC, head and neck cancer.

A high CONUT score was significantly associated with worse OS and DFS (50). Both outcomes were supported by low quality evidence, with low to moderate heterogeneity (I^2^ = 26.1% for OS and 45.6% for DFS) (Supplementary Table S2, Figure 11).

##### 3.2.1.7 Other tumors not classified by organ system

**Figure 12:**
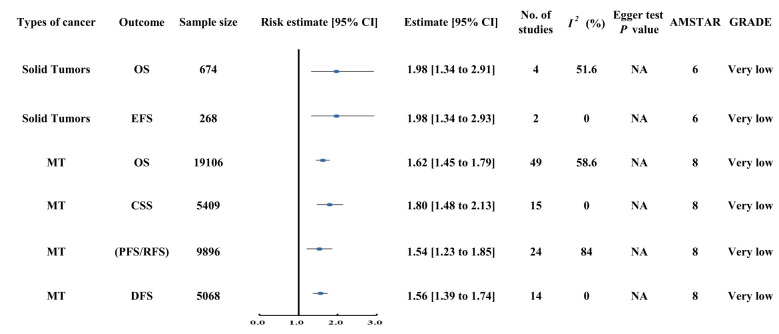
Forest plot for pooled associations between elevated CONUT score and long-term survival in unclassified solid and overall malignant tumors supported by very low quality evidence. Rows list cancer subtype, survival endpoint, sample size, study count, I^2^, Egger's P, AMSTAR score, and GRADE rating. CI, confidence interval; AMSTAR, a measurement tool to assess systematic reviews; I^2^, inconsistency index (I^2^ statistic); Egger test, Egger's regression test; GRADE, grading of recommendations assessment, development, and evaluation; NA, not available; OS, overall survival; EFS, event-free survival; PFS, progression-free survival; DFS, disease-free survival; RFS, recurrence-free survival; CSS, cancer-specific survival; MT, malignant tumors.

In pooled analyses of solid tumors (51) and overall malignant tumors (28), elevated CONUT scores were associated with poorer OS, EFS, CSS, DFS, and PFS/RFS. All these findings were graded as very low quality evidence, with between-study heterogeneity ranging from 0% to 84% (Supplementary Table S2, Figure 12).

##### 3.2.1.8 Glioblastoma

A meta-analysis of three retrospective cohort studies (490 patients total) showed that a higher CONUT score was associated with poorer OS (HR = 2.79, 95% CI: 2.01–3.89) (52). This association was supported by low quality evidence with moderate between-study heterogeneity (I^2^ = 39%).

#### 3.2.2 For perioperative outcomes

##### 3.2.2.1 Postoperative complications

**Figure 13:**
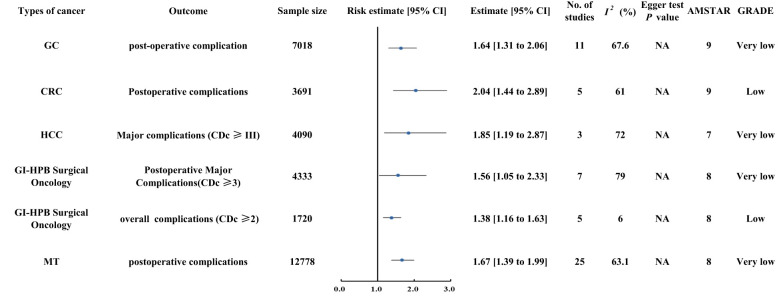
Forest plot for pooled associations between elevated CONUT score and postoperative complication risk across multiple malignancies supported by low or very low quality evidence. Rows list cancer subtype, survival endpoint, sample size, study count, I^2^, Egger's P, AMSTAR score, and GRADE rating. CI, confidence interval; AMSTAR, a measurement tool to assess systematic reviews; I^2^, inconsistency index (I^2^ statistic); Egger test, Egger's regression test; GRADE, grading of recommendations assessment, development, and evaluation; NA, not available; CDc, Clavien–Dindo classification; HCC, hepatocellular carcinoma; CRC, colorectal cancer; GC, gastric carcinoma; GI-HPB Surgical Oncology, gastrointestinal and hepatopancreatobiliary surgical oncology; MT, malignant tumors.

Elevated CONUT score was associated with an increased risk of postoperative complications across multiple malignancies, including GC, CRC, HCC (major complications, Clavien–Dindo ≥ III), and GI-HPB cancer surgery (major and overall complications) (11, 13, 18, 20). However, heterogeneity was moderate to high (I^2^ = 6%−79%), and evidence quality was predominantly low or very low (Supplementary Table S2, Figure 13).

##### 3.2.2.2 Postoperative mortality

As noted in Section 3.1.2, high CONUT scores were strongly associated with increased postoperative mortality in patients with MTs and those undergoing GI-HPB cancer surgery (20, 28). These were the only perioperative outcomes supported by moderate quality evidence, with no between-study heterogeneity (I^2^ = 0%).

### 3.3 Inconsistent or non-significant findings

**Figure 14:**
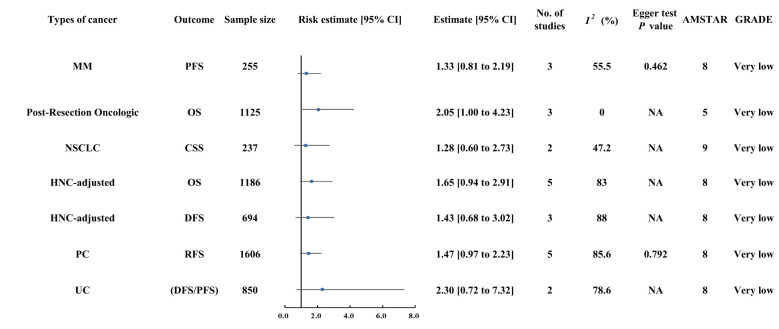
Forest plot for pooled associations between elevated CONUT score and survival endpoints for non-significant findings supported by very low quality evidence. Rows list cancer subtype, survival endpoint, sample size, study count, I^2^, Egger's P, AMSTAR score, and GRADE rating. CI, confidence interval; AMSTAR, a measurement tool to assess systematic reviews; I^2^, inconsistency index (I^2^ statistic); Egger test, Egger's regression test; GRADE, grading of recommendations assessment, development, and evaluation; NA, not available; OS, overall survival; PFS, progression-free survival; DFS, disease-free survival; RFS, recurrence-free survival; CSS, cancer-specific survival; MM, multiple myeloma; UC, urological cancer; NSCLC, non-small cell lung cancer; PC, pancreatic cancer; HNC, head and neck cancer.

Not all outcomes were statistically significant. A total of 7/86 (8.1%) outcome measures showed no significant association between CONUT score and prognosis, all rated very low quality evidence and accompanied by substantial between-study heterogeneity, including: PFS in MM (40); OS in postoperative oncological settings (53); CSS in untreated NSCLC (54); Adjusted OS and PFS in HNC (55); RFS in PC (56); and DFS/PFS in UC (37) (Supplementary Table S2, Figure 14).

### 3.4 CCA analysis of study overlap

To quantify primary study overlap across the included meta-analyses, we calculated the CCA. The 40 meta-analyses cited 447 references across 210 unique primary studies, yielding slight overlap (CCA = 2.89%, within the 0–5% range). The full citation matrix (210 × 40) is provided in Supplementary Table S3. However, because the overall CCA may mask substantial heterogeneity in primary study overlap across different cancer types, we performed stratified CCA analyses by major cancer category. Moderate overlap was found for digestive (5.8%) and gynecological (9.1%) malignancies. By contrast, hematologic (32.6%), respiratory (60.0%), urologic (60.0%), and head and neck (87.5%) cancers presented very high overlap. These stratified findings indicate that, despite low overall overlap, several cancer subgroups featured substantial primary study duplication—mostly those with a limited number of constituent meta-analyses—potentially impairing the independence of the synthesized evidence for these disease subgroups. Accordingly, conclusions drawn for hematologic, respiratory, urologic, and head and neck cancers should be interpreted with particular caution, as the very high primary study overlap may inflate the apparent consistency of the findings.

### 3.5 Heterogeneity of included studies

Of the 86 outcome measures analyzed in this study, 41 (47.6%) demonstrated significant heterogeneity (I^2^ > 50%). This heterogeneity in the majority of outcomes can be attributed to several potential factors, including study setting, gender, age, study quality, sample size, disease stage and subtype, treatment regimen, follow-up duration, inconsistent cutoff values for CONUT scores, and adjustments for confounding variables.

### 3.6 Publication bias of included studies

Among the 86 outcome measures analyzed, Egger's test results were available for only 29 (33.7%), of which 9 (31%) showed significant publication bias (*P* < 0.05). For outcome measures without available Egger's test results, publication bias was assessed by examining funnel plots, and these assessments were incorporated into the GRADE evaluation. Furthermore, 13 of the 40 included meta-analyses (32.5%) did not perform any assessment of publication bias for any outcome measure (Supplementary Tables S2, S4).

### 3.7 AMSTAR and GRADE evaluation of included studies

Most of the included meta-analyses achieved high AMSTAR scores for reporting standards: 30 of the 40 included studies (75%) attained an AMSTAR score of 8 or out of 11 points. Detailed AMSTAR scores for all included meta-analyses are provided in Supplementary Table S1. This study employed the GRADE classification system to evaluate the evidence quality of the 86 included outcome measures. The results showed that the vast majority of outcome measures were rated as “low” (*n* = 25) or “very low” (*n* = 51) in quality, accounting for 76 of 86 outcomes (88.4%). Only 10 outcome measures (11.6%) were rated as “moderate” quality. Notably, no “high” quality evidence was identified in this umbrella review (Supplementary Tables S2 and S3). The 10 moderate-quality evidence outcomes included the following cancer types and endpoints: esophageal cancer (CSS) (25); gastrointestinal cancer (OS and CSS) (19); colorectal cancer (OS and CSS) (13, 15); bladder cancer (OS and RFS) (26); preoperative lung cancer (DFS) (27); and postoperative mortality in patients with malignant tumors (28) as well as those undergoing GI-HPB cancer surgery (20).

## 4 Discussion

This umbrella review systematically synthesized data from 40 meta-analyses investigating the association between the CONUT score and cancer prognosis, with a secondary synthesis of 86 outcome measures derived from the published pooled estimates of these included meta-analyses. Of these, 79 were statistically significant.

The findings of this review indicate that an elevated CONUT score is significantly associated with poor prognosis across multiple cancer types, including digestive, urinary, hematological, respiratory, gynecological, and head and neck cancers, as well as glioblastoma. The ranges of HRs, ORs, and RRs are presented in the Results section. It is important to note that HR, RR, and OR are distinct effect measures with different statistical interpretations and cannot be directly quantitatively compared: HR estimates instantaneous event risk over time, RR estimates the risk ratio between two exposure groups, and OR estimates the odds ratio, with each metric corresponding to specific study designs and analytical frameworks.

Tumors of the digestive system accounted for the largest proportion (40%) of the 40 included meta-analyses. Despite the consistent significant associations observed, the prognostic value of the CONUT score was substantially limited by the overall low quality of the available evidence. Specifically, 88.4% (76/86) of the included outcome measures were rated as low or very low quality, only 10 outcomes were assessed as moderate quality, and no high quality evidence was identified. This finding highlights the inherent limitations of existing studies, including significant between-study heterogeneity (47.6% of the 86 outcomes had an I^2^ > 50%), potential publication bias (31% of Egger's tests yielded *P* < 0.05), and a lack of standardization in CONUT cutoff values and confounding factor adjustment—all of which further weakened the strength of the available evidence.

In this umbrella review, ten outcome measures were assessed as providing moderate-quality evidence across multiple cancer types and clinical contexts, as detailed in the Results section. These indicators were generally characterized by relatively large effect sizes, acceptable heterogeneity (predominantly I^2^ < 50%), and the high methodological quality of the included meta-analyses (AMSTAR score ≥8 out of 11). Higher AMSTAR scores primarily reflect improved standardization of reporting and procedural rigor, yet they cannot overcome the inherent limitations of the original observational studies, including constraints in study design and confounder control. A high AMSTAR score is therefore a necessary, but not sufficient, condition for establishing high clinical reliability. Consequently, the prognostic associations of the CONUT score are more reliably observed within methodologically rigorous frameworks. In terms of evidence grading, moderate-quality evidence implies moderate confidence that the true effect approximates the pooled estimate, although future high-quality research may alter current conclusions. Nonetheless, cautious interpretation remains warranted, and the CONUT score should not be employed as a standalone predictive biomarker for treatment selection. Whether it may serve as an adjunctive risk stratification marker requires further validation in prospective studies. In this umbrella review, a “prognostic biomarker” is defined as a factor associated with long-term survival outcomes (e.g., OS, PFS) independent of treatment allocation. The term “predictive biomarker” is not adopted in this review, as none of the included meta-analyses evaluated treatment-specific responses. The CONUT score has been investigated as a potential risk stratification marker, but this application remains to be validated.

Regarding short-term perioperative outcomes (as shown in Results Section 3.2.2), this umbrella review indicates that an elevated CONUT score may be associated with postoperative complications and mortality across various malignant tumors. Nonetheless, several important limitations should be noted. First, the independent and incremental prognostic value of the CONUT score for postoperative mortality—beyond that provided by traditional surgical risk models—remains unvalidated. Second, the number of primary studies included for this endpoint was relatively small. As such, the observed I^2^ value of 0% may reflect limited sample sizes and insufficient statistical power, rather than true clinical or methodological homogeneity. Although moderate-quality evidence supports an association between elevated CONUT scores and postoperative mortality, these limitations warrant cautious interpretation of the findings. Future studies should incorporate the CONUT score into multivariable clinical prediction models to evaluate its incremental prognostic value in the perioperative setting.

In this umbrella review, 7 out of 86 outcomes (8.1%) did not reach statistical significance (see Results, Section 3.3). The coexistence of both significant and nonsignificant results across cancer subtypes and treatment modalities reflects the heterogeneous prognostic performance of the CONUT score, underscoring a need for pooled synthesis to avoid overstating the consistency of the evidence. These null findings, although mostly graded as very low certainty, further highlight this heterogeneity and reduce the apparent consistency of the evidence across different cancer types and clinical settings. The reasons for these nonsignificant findings remain unclear because of the limitations of the available data, including small sample sizes, inconsistent cutoff values, and inadequate adjustment for confounders. Importantly, these null results should not be interpreted as definitive evidence against the prognostic utility of the CONUT score, nor should they be confidently attributed solely to insufficient statistical power without direct supporting evidence. Multiple explanations may underlie these inconsistent observations. First, the prognostic information captured by the CONUT score may partially overlap with established risk factors such as tumor stage, performance status, and treatment modality; when these covariates are statistically adjusted, the independent contribution of CONUT may be attenuated, as illustrated by analysis of HNC in which unadjusted estimates were significant but adjusted ones were not (55). Second, biological differences across cancer types—whereby baseline nutritional status may be masked by treatment-related factors or disease aggressiveness, as suggested by null findings in postoperative settings and untreated NSCLC (54)—may further influence prognostic relevance. Third, methodological heterogeneity across meta-analyses, including variability in CONUT cut-off values, assessment timing, outcome definitions, and confounder adjustment, likely compromises cross-study comparability and may yield inconsistent conclusions even within the same cancer type. Fourth, non-significant findings may reflect limited statistical power due to small sample sizes or low event counts rather than a true absence of prognostic value. Collectively, these observations indicate that nonsignificant results should not be interpreted as definitive evidence against the utility of the CONUT score. Instead, they highlight the importance for standardized cutoff thresholds, consistent assessment timing, rigorous confounder adjustment, and deeper exploration of the biological mechanisms underlying cancer type-specific effects in future research.

Malnutrition is a common complication in patients with cancer, which can be caused by the tumor itself or by surgery, radiotherapy, chemotherapy, and other anti-tumor treatments (57). Cancer-related malnutrition can adversely affect quality of life, treatment tolerance, and clinical outcomes in patients with cancer (58–60). According to relevant studies, approximately 10% to 20% of cancer patients ultimately die from malnutrition-related complications rather than from the tumor itself (61, 62). ESPEN guidelines strongly recommend that all patients with cancer undergoing anticancer treatment and those with an expected survival of more than 3 to 6 months should undergo regular nutritional risk screening (63). For patients identified as being at nutritional risk, a comprehensive assessment of potentially treatable contributing factors is required (64). Nutritional risk assessment is an important component of clinical management and prognostic evaluation. A recent umbrella review by Li et al. (65) systematically evaluated the associations between eight malnutrition diagnostic tools and clinical outcomes in patients with cancer. The findings indicated that the Prognostic Nutritional Index (PNI), Geriatric Nutritional Risk Index (GNRI), and CONUT score were the three assessment tools with the highest evidence ratings and were strongly recommended (Class II).

PNI, GNRI, and the CONUT score are nutritional prognostic biomarkers that all incorporate serum albumin but differ in their components, target populations, and clinical applications. The PNI combines albumin and lymphocyte count to assess surgical risk and nutritional-immune status, with its prognostic value across multiple cancer types supported by moderate-quality evidence from a recent umbrella review (66). The GNRI, calculated using albumin and BMI, is specifically designed for elderly patients, accounting for age-related physiological changes to evaluate nutrition-associated complications and mortality risk (67). The CONUT score integrates three routinely measured parameters—albumin, lymphocyte count, and total cholesterol—into a composite score (range 0–12; higher scores indicate worse nutritional status). Originally developed for hospitalized patients, it has since been widely adopted in oncology, in part due to its simplicity and the fact that it relies on routine laboratory tests with no need for additional procedures (1, 68). Multiple studies have demonstrated its prognostic relevance across diverse cancer types. For instance, the CONUT score was superior to PNI in predicting survival in CRC (69), served as an independent prognostic factor for OS in elderly patients undergoing resection for NSCLC (70), and in RCC (71), and helped identify gastric cancer patients unlikely to benefit from immune checkpoint inhibitor therapy (72). It was also an independent prognostic indicator following esophagectomy for EC (73) and exhibited superior predictive efficacy for all-cause mortality among five screening tools evaluated in PC (74).

However, conflicting evidence persists. Kim et al. reported that PNI outperformed CONUT in predicting outcomes in stage I–III colorectal cancer (4), and a low GNRI was significantly associated with worse OS and PFS in HNC, whereas the CONUT score showed no significant association with either endpoint after adjustment for confounders including TNM stage and ECOG performance status (55). Such discrepancies may stem from variations in confounder adjustment, CONUT cutoff values, and heterogeneity in sample sizes and study designs. Given the absence of head-to-head studies directly comparing the predictive performance of these tools—using metrics such as the C-index and net reclassification improvement—within identical patient cohorts, no single tool can be identified as superior. Selection should therefore be guided by cancer type, clinical setting (e.g., GNRI may be particularly suitable for older adults), and available laboratory parameters. Prospective head-to-head investigations are warranted to clarify their comparative utility and to optimize nutritional risk stratification in oncology.

The CONUT score comprises three key indicators: serum albumin, total lymphocyte count, and total cholesterol, which respectively reflect the body's nutritional status, immune function, and metabolic level. Consequently, the association between the CONUT score and tumor prognosis may be partly explained by the synergistic impact of nutritional deficiency, immune suppression, and metabolic disorders on the tumor microenvironment. First, serum albumin level is a biomarker of systemic inflammation and nutritional status. Tumor-associated inflammatory responses may exacerbate energy expenditure in malnourished individuals, potentially perpetuating a cycle of inflammation and malnutrition that may influence tumor progression (75). Second, lymphocytes play an essential role in inhibiting tumor cell proliferation and metastasis via immune pathways (76). Peripheral blood lymphocyte count serves as an indicator of systemic antitumor immune capacity, and its reduction may signify impaired immune surveillance (77, 78). Third, cholesterol has been implicated in tumor development and immune evasion (79). Low serum cholesterol levels have been associated with increased tumor incidence and mortality (80, 81), a finding observed in patients with lung cancer (82), gastrointestinal cancer (83), thyroid cancer (84), and hematological malignancies (85). Together, serum albumin, total lymphocyte count, and total cholesterol characterize the nutritional, immune, and metabolic status of cancer patients from multiple perspectives. This integrated profile may form the biological basis that enables the CONUT score to reflect cancer prognosis. Disruption of this regulatory network may facilitate tumor progression and reduce patient tolerance to surgical interventions, providing a potential biological rationale for the observed association between elevated CONUT scores and adverse perioperative outcomes.

Although these mechanistic insights support the biological plausibility of the CONUT score as a prognostic biomarker, all evidence summarized in this umbrella review originates from observational studies, which cannot confirm causality or definitive independent predictive effects. A high CONUT score primarily acts as a comprehensive marker reflecting an adverse systemic baseline status, which is driven by tumor aggressiveness, systemic inflammation, malnutrition, and comorbidities. Therefore, the respective contributions of the CONUT score's inherent prognostic value and its surrogate role for poor systemic conditions to prognostic outcomes remain unclear. Large-scale prospective studies with rigorous adjustment for confounding variables are warranted to clarify this issue. Until its independent incremental prognostic value is fully validated, the CONUT score should be considered an auxiliary marker with prognostic relevance rather than a confirmed causal target for clinical intervention.

## 5 Advantages

This study utilized an umbrella review methodology to synthesize 40 meta-analyses that examine the relationship between the CONUT score and cancer prognosis. These meta-analyses included 111 outcome measures related to malignancies of the digestive, urinary, hematological, respiratory, and other systems. To the best of our knowledge, this is the first umbrella review to systematically synthesize the prognostic evidence of the CONUT score across multiple cancer types, as well as its prognostic value for perioperative outcomes. By performing a secondary synthesis of published pooled estimates, the study addresses the inherent limitations of individual meta-analyses or primary studies, such as restricted evidence coverage and challenges in interpreting heterogeneity, thereby offering more robust evidence-based support for clinical practice.

The study rigorously followed standardized systematic review methodologies. It was registered with PROSPERO to mitigate research bias, and the search strategy was developed in accordance with PRISMA-S guidelines. We calculated the CCA to quantify overlapping primary studies across included meta-analyses and evaluate the independence of the synthesized evidence. The AMSTAR and GRADE tools were used to evaluate study quality and determine the level of confidence in the effect estimates.

## 6 Limitations

Several limitations of this study should be acknowledged. First, 88.4% (76/86) of the evidence is rated as low or very low quality, with only 10 outcomes of moderate quality and none of high quality. This significantly constrains the robustness of any recommendations derived from our findings. Second, substantial heterogeneity is identified in 47.6% of the outcome indicators. Egger's test indicated publication bias in 31% of cases, and some studies did not assess publication bias at all. The absence or incompleteness of publication bias assessment limits the quality of the evidence. Failure to detect or report publication bias may have omitted negative results, thereby biasing our estimates of the CONUT score's prognostic value upward. Consequently, this further undermines our ability to draw definitive conclusions. Third, the original studies lacked standardized CONUT cutoff values (a major barrier to clinical implementation) and adequate adjustment for confounding factors. Cutoff values varied considerably (from ≥ 2 to ≥ 5), with no consensus on the optimal threshold for specific cancer types or clinical settings. This variability directly affects the comparability and generalizability of the findings. Fourth, some subgroup analyses involved overlapping patient populations without appropriate correction, potentially introducing statistical bias. Fifth, the absence of high-quality prospective interventional studies limits our ability to evaluate the CONUT score's clinical value.

Beyond the methodological flaws in the existing evidence, our study has inherent analytical limitations. Given the evaluation of multiple cancer types and outcomes, the biological and statistical correlation among endpoints (e.g., OS, PFS, and DFS), treating each significant finding as independent evidence may lead to overinterpretation of the CONUT score's prognostic value. Therefore, the findings of this study should be considered exploratory—intended to map the evidence landscape rather than to draw definitive conclusions.

We also acknowledge several methodological constraints of this review. First, the inclusion criteria were restricted to English-language meta-analyses, which introduces potential language bias and may exclude valuable studies published in other languages. Second, gray literature (e.g., conference abstracts and dissertations) was not included, potentially omitting pertinent findings. Third, by focusing exclusively on meta-analyses rather than primary studies, we could not directly assess the methodological quality of the original research. Thus, this may indirectly affect the reliability of our conclusions.

Several limitations of the CCA assessment should be acknowledged. First, the CCA relies on complete reference reporting by the included meta-analyses; the omission of non-English or gray literature studies may slightly underestimate the true overlap. Second, the overall CCA of 2.89% masks substantial within-cancer overlap: the CCA ranged from 32.6% to 87.5% for head and neck, respiratory, urinary, and hematological malignancies, severely compromising evidence independence. The pooled findings for these cancer categories should be interpreted with particular caution, as the apparent consistency of the results may be partly driven by overlapping primary studies rather than independent replication. Third, CCA quantifies only citation overlap and cannot identify overlapping patient cohorts across distinct primary studies, which is an inherent limitation of citation-matrix-based umbrella reviews. Fourth, subgroups with extremely high CCA (e.g., 87.5% for HNC) contained few meta-analyses (c = 2), thereby magnifying the influence of individual overlapping trials. Fifth, sensitivity analyses were not performed: the included meta-analyses reported only aggregated summary statistics, and individual patient data or study-level effect sizes were not available for recalculation. Consequently, we could not quantify the precise impact of primary study overlap on the pooled effect estimates. Despite these limitations, the low overall CCA of 2.89% supports the acceptable independence of the entire evidence base, with stratified subgroup analyses enabling transparent domain-specific interpretation.

## 7 Summary

The available evidence, predominantly of low to very low certainty (76/86 outcomes, 88.4%), suggests that an elevated CONUT score is associated with poor prognosis across multiple cancer types. Moderate-certainty evidence supports this association in ten specific contexts—including colorectal cancer, bladder cancer, and postoperative mortality in malignant tumors and GI-HPB surgery—indicating potential prognostic relevance. However, the current evidence base is limited by substantial heterogeneity, inconsistent CONUT cutoff values, inadequate confounder adjustment, and a lack of high-certainty studies. Importantly, all included evidence originates from observational studies; therefore, the observed associations do not establish causality or independent predictive utility of the CONUT score. Consequently, routine clinical use of the CONUT score as an independent decision-making tool is not currently warranted. The CONUT score may serve as an adjunctive risk stratification marker to complement clinical judgment, but its role should be further validated in well-designed prospective studies that rigorously adjust for established confounders and directly compare its incremental value against existing nutritional assessment tools.

## Data Availability

The original contributions presented in the study are included in the article/supplementary material, further inquiries can be directed to the corresponding author.
